# The effects of the miR-21/SMAD7/TGF-β pathway on Th17 cell differentiation in COPD

**DOI:** 10.1038/s41598-021-85637-0

**Published:** 2021-03-18

**Authors:** Shengyang He, Shenghua Sun, Junjuan Lu, Lili Chen, Xiang Mei, Liqiu Li, Zhengpeng Zeng, Mubin Zhong, Lihua Xie

**Affiliations:** Department of Pulmonary and Critical Care Medicine, The Third Xiangya Hospital of Central South University, Changsha, China

**Keywords:** Immunology, Immunological disorders, Lymphoproliferative disorders

## Abstract

Chronic obstructive pulmonary disease (COPD) is a complex disease with multiple etiologies, while smoking is the most established one. The present study investigated the modulation of T-helper 17 (Th17) cell differentiation by the miR-21/Smad7/TGF-β pathway, and their roles in COPD. Lung tissues were obtained from lung cancer patients with or without COPD who underwent lobotomy and the levels of miR-21, TGF-β/Smad signaling molecules, RORγT, and other Th17-related cytokines were detected. Mouse COPD models were built by exposing both wild-type (WT) and miR-21^−/−^ mice to cigarette smoke (CS) and cigarette smoke extract (CSE) intraperitoneal injection. Isolated primary CD4^+^ T cells were treated with either CS extract, miR-21 mimics or inhibitors, followed by measuring Th17 cells markers and the expression of TGF-β/Smad signaling molecules and RORγT. Increased levels of miR-21, Smad7, phosphorylated (p)-Smad2, p-Smad3, TGF-β, and Th17-related cytokines was detected in the lungs of COPD patients. Lung function in modeled WT mice, but not miR-21^−/−^ ones, deteriorated and the number of inflammatory cells in the lung tissues increased compared to the control WT-mice. Moreover, primary CD4^+^ lymphocytes tend to differentiate into Th17 cells after the treatment with CSE or miR-21 mimics, and the expression of RORγT and the TGF-β/Smad signaling were all increased, however miR-21 inhibitors worked reversely. Our findings demonstrated that Th17 cells increased under COPD pathogenesis and was partially modulated by the miR-21/Smad7/TGF-β pathway.

## Introduction

Chronic obstructive pulmonary disease (COPD) is one of the most common causes of morbidity and mortality worldwide^1^. COPD is characterized by persistent and irreversible airflow limitation that is usually progressive^2^. No effective treatments are currently exist to stop the development of COPD and this is largely due to the fact that the pathogenic mechanisms are still not fully understood.

Numerous studies have shown that cigarette smoking can induce infiltration of inflammatory cells into the lung^3^.We found a massive infiltration of inflammatory cells in the lungs of COPD mice^4^. Among the inflammatory cells, a subset of CD4^+^ T lymphocytes, called T-helper 17 (Th17) cells, produce interleukin-17A (IL-17A) which further contributes to increased secretion of various inflammatory cytokines by other cells. IL-17A is a key cytokine in the rat elastase-induced emphysema model. Th17 cells could further give rise to the persistent inflammation in lungs of COPD patients^5^, resulting in uncontrollable airway and alveolar inflammation, damaging the lung tissue structures, and eventually leading to emphysema^3,6^. However, the mechanisms behind Th17 cell differentiation are yet to confirm.

MicroRNAs (miRs) are endogenous non-coding RNAs approximately 22 nucleotides in length that can regulate target gene expression at the post-transcriptional level. MiRs participate in many pulmonary diseases, including lung cancer, COPD, asthma, and idiopathic pulmonary fibrosis (IPF)^7^. However, the exact roles of miRs in COPD have not been sufficiently determined. Our group previously found that miR-21 expression was notably increased in the COPD rats, and we reconfirmed this result in the peripheral serum, mononuclear cells and circulation exosomes of COPD patients^8,9^. Some recently published studies reported that miR-21 could promote differentiation of CD4^+^ cells into Th17 cells in autoimmune encephalomyelitis and viral myocarditis^10,11^. We therefore hypothesized that miR-21 also affects Th17 cell function in COPD patients.

Smad proteins are members of the transforming growth factor-β (TGF-β) superfamily, and participating in many physiological functions^12^. One study found that TGF-β affects the activity of many immune cells and its overexpression enhanced Th17 cell differentiation^13^.

Therefore, we proved the hypothesis that miR-21 plays a role in the pathogenesis of COPD by affecting Th17 cells functions through modulating the Smad7/TGF-β pathway.

## Materials and methods

### Collection of human lung tissues

Lung tissues were collected from non-smoker patients both without COPD and with COPD who were undergoing pulmonary lobectomy due to lung cancer at the Xiangtan Central Hospital (Hunan, P.R. China). Healthy lung tissue at least 2 cm away from the tumor was also collected. Lung tissues of COPD patients were also collected from patients who underwent pulmonary lobectomy due to intractable pneumothorax or pneumatocele. COPD diagnoses were made according to GOLD2017^2^. Patients who had other chronic pulmonary diseases, such as bronchiectasia, asthma, tuberculosis, interstitial lung disease, or pneumosilicosis, were excluded. All collected lung tissues were stored at − 80 ℃ until further analysis.

The present study was approved by the Institutional Human and Animal Care Ethics Committee at the Third Xiangya Hospital of Central South University and Xiangtan Central Hospital, in accordance with the principles of the Helsinki Declaration II. Written informed consent was obtained from all patients prior to their enrollment into the study. All methods were carried out in accordance with relevant guidelines and regulations and the study was carried out in compliance with the ARRIVE guidelines.

### Enzyme-linked immunosorbent assay (ELISA)

Expression of Th17 related cytokines [interleukin (IL)-17A, IL-6, IL-1β, IL-21, IL-22, tumor necrosis factor-α (TNF-α), and IL-10] in human lung tissue were detected using the multi-factor ELISA kit (ExCell Biology, Inc. Shanghai, China) according to the manufacturer’s instructions.

### Real-time quantitative polymerase chain reaction (RT-qPCR)

Total RNA from human lung tissue was isolated using the UNIQ-10 column Trizol RNA isolation kit (SangonBiotech, Shanghai, China). The PrimeScript RT reagent Kit (Takara Bio Inc., Shiga, Japan) was utilized for reverse transcription. Relative mRNA levels were quantified using two-step quantitative real-time polymerase chain reaction (PCR; Applied Biosystems, Carlsbad, CA, USA) with the SYBR Green PCR Master Mix (BioRad, Laboratories Inc., Hercules, CA, USA). U6 and GAPDH were utilized as internal controls. Primer for miR-21 is Bulge-Loop (RiboBio, Guangzhou, China). All other primers were designed by Primer Premier 5.0 (Premier Biosoft international, Palo Alto, CA, USA), and see Table 1.

**Table 1:** Primer sequences.

Primers	Sequences
TGF-β	F: agcaacaattcctggcgatacctc R: tcaaccactgccgcacaactc
SMAD7	F:aatggcttttgcctcggacagg R: cacaaagctcatgtgcacggtc
RORγT	F:tgcgactggaggaccttctac R:tcacctcctcccgtgaaaag

### Mouse COPD model building

Eight-week-old C57BL/6 wild-type (WT) mice (Vital River Laboratory Animal Technology Co., Ltd, Beijing, China, n = 5) and the miR-21^−/−^ mice (University of Texas, Southwestern Medical Center, TX, USA, n = 5) were enrolled in the present study. The WT mice were divided into a control group and a COPD model group, respectively. The COPD group was built as previously described by our group^4^. Briefly, mice were exposed to CS in a sealed box with a ventilation hole for two cycles per day and 28 days in total, except for days 1, 12, and 23, when mice were given the intraperitoneal injection of 100% cigarette smoke extract (CSE) solution (0.3 ml/20 g). The control group mice were maintained in fresh air with no special treatment and on days 1, 12, and 23 they were given an intraperitoneal injection of PBS (0.3 ml/20 g).

### IHC staining

Paraffin embedded lung tissue sections underwent IHC to detect RORγT (Abcam, UK, dilution 1:100). Primary antibodies were incubated overnight at 4 °C. Secondary antibodies are horseradish peroxidase-conjugated, being incubated for 15 min at 37 °C. The target proteins were visualized using diaminobenzidine (DAB, Sigma-Aldrich Co., USA). Five lung tissue sections from each mouse that underwent a complete lung function test were stained and five 400× fields from each section were randomly chosen for further analyses.

### Western blot

Expression of SMAD7, phosphorylated (p)-SMAD2, RORγT, and TGF-β were detected in both the human lung tissue homogenates samples and in the sorted CD4^+^ mouse lymphocytes using standard Western blot techniques. Isolated proteins were transferred onto a nitrocellulose membrane, and immunoblotting was performed using mouse monoclonal anti-SMAD-7 (R&D Systems, USA, dilution 1:1000), anti-p-SMAD2 (Abcam, USA, dilution 1:1000), anti-p-SMAD3 (Abcam, USA, dilution 1:1000), anti-TGF-β (Abcam, USA, dilution 1:1000), anti-ROR-γT (Abcam, USA, dilution 1:1000), anti-β-actin (Proteintech, USA, dilution 1:5000), and conjugated HRP-labeled secondary mouse or rabbit antibodies (Proteintech, USA). Images were captured using the Quantity One Analysis Software (Bio-Rad Laboratories Inc, Hercules, CA, USA).

### Lung function tests

We used an unrestrained whole-body plethysmography (PLY3211; Buxco Research Systems, Wilmington, NC, USA) provided by the School of Basic Medical Science of Central South University (Changsha, China) to measure lung function of all mice before sacrifice. Data of Raw (Resistance of airway), PEF (Peak Expiratory Flow rate), Cdyn (Lung Dynamic Compliance), and Ti/Te (Inspiratory Time/Expiratory Time)tests were collected for further analyses.

### Preparation of cigarette smoke extract (CSE)

CSE was obtained by burning non-filter Furong cigarettes (tar: 13 mg, nicotine: 1.0 mg, carbon monoxide: 14 mg/cigarette; China Tobacco Hunan Industrial Co. Ltd., Changsha, China). Smoke from each cigarette was dissolved in 10 ml phosphate buffered saline (PBS) to obtain 100% CSE. Before injection, bacteria and particles were removed from the CSE solution using a 0.22-µm pore filter (Thermo Fisher Scientific, Waltham, MA, USA). The solution was utilized within 30 min after preparation.

### Morphological changes of lung tissue

Morphological changes of lung tissues in the COPD group and control group were evaluated as previously described after H&E staining^14^. Briefly, we used Image-Pro plus 6.0 to measure the mean alveolar septal thickness (MAST), mean linear intercept (MLI), and destructive index (DI). Inflammation pathology scores were obtained by combining the degree of peri-bronchial inflammation and lymphocytes in the exudate, along with the exudate in the airway lumen^15^.

### Magnetic cell sorting (MACS) of CD4^+^ cells

The spleens and lymph glands of mice in the COPD group and control group were collected and ground in PBS and then filtered through a 70 μm filter. The cell suspension was collected and treated with red blood cell lysis buffer (Cwbiotech, China). After a 3 min incubation at 4 ℃, cells were washed twice with PBS. CD4^+^ cells were separated by incubating 1 × 10^7^ cells with 90 μl magnetic bead buffer (Miltenyi, Germany) and 10 μl of a magnetic CD4^+^ bead antibody (Miltenyi, Germany) at 4 ℃ in the dark. The cells were then sorted using a MACS classifier (Miltenyi, Germany) according to the operating instructions.

### Th17 cell differentiation

The MACS-sorted CD4^+^ cells were divided into four equal groups—the control group, the CSE group, the miR-21 mimics (Qiagen, Germany, concentration: 5 nM) group, and the miR-21 inhibitor (Qiagen, Germany, concentration: 50 nM) group. Briefly, 1 × 10^6^ cells were seeded in each well of a 6-well plate (coated with CD3 antibody, 2 μg/ml, BD Pharmingen) with a number of differentiation factors, including CD28 (2 μg/ml, BD Pharmingen), TGF-β (1 ng/μl, ExCeLL Biology, Shanghai, China), IL-6 (50 ng/ml, Proteintech, USA), and IL-1β (2 ng/ml Proteintech, USA). After 48 h of incubation, CSE, miR-21 mimics, and miR-21 inhibitors were added, respectively. After another 48 h, cells were incubated with an IL-17A antibody for flow cytometry detection of Th17 cells. Before flow cytometry, cells were treated with O-tetradecanoylphorbol-13-acetate (PMA) for 5 h. Differentiated Th17 cells were collected for total RNA and proteins isolation.

### Statistical analysis

All data were analyzed using the SPSS 22.0 statistical software (IBM Corporation, Armonk, NY, USA) and GraphPad Prism 5 software (San Diego, CA, USA). Data are presented as the mean ± standard error of the mean (SEM). Comparisons between two groups of data were performed using the unpaired two-tailed Student’s *t* test and ANOVA plus multiple comparison. All data were verified normal distribution. A P-value of less than 0.05 was considered statistically significant.

## Results

### Upregulation of miR-21 and Th17 cells level in COPD patients

12 patients (6 non-smoker and 6 COPD patients) were enrolled. There were no significant differences in the distribution of age, gender, or BMI between the two groups. The smoking index and lung function parameters (FEV1 and FEV1/FVC%) in the COPD patients were significantly different compared to the non-smoking controls (P < 0.05, Table 2).

**Table 2:** The demographic and clinical characteristics of enrolled patients.

	Non-smoking control	COPD patients
Gender M/F	6/0	6/0
Age (years)	57.33 ± 3.20	59 ± 9.76
BMI (kg/m^2^)	19.85 ± 3.02	20.65 ± 1.94
Smoking index (pack-years)	0	25 ± 5.48*
FEV1 (%pred)	93.88 ± 3.27	60.33 ± 5.17*
FEV1/FVC%	87.58 ± 4.08	61.52 ± 4.06*

Data are expressed as median and range.

*P < 0.05 versus non-smoking controls.

**Figure 1:**
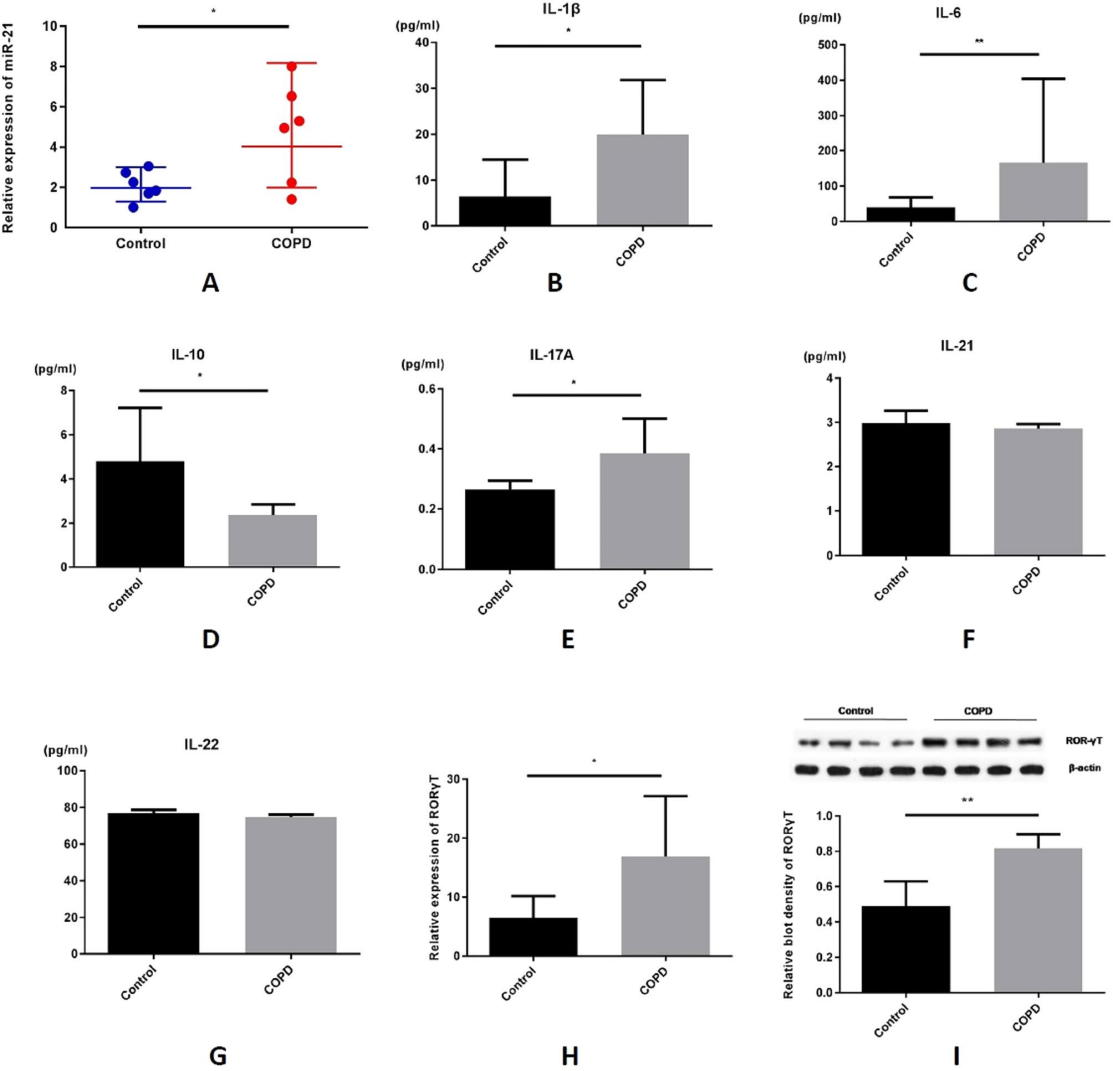
MiR-21 and Th-17 related cytokines levels increase in lungs of COPD patients. (**A**) COPD patients have a higher level of miR-21 in lungs. (**B**)–(**G**) Th17-related cytokines (IL-1β, IL-6, IL-10, IL-17A, IL-21, IL-22, respectively) are all increased in lungs of COPD patients. (**H**) The mRNA level of RORγT increases in lungs of COPD patients. (**I**) The protein level of RORγT increases in lungs of COPD patients. In western blot, different samples of the same marker are from the same blot. The data are expressed as the mean ± SEM (n = 6 in each group), t-test was applied in this part, *P < 0.05.

We found that the relative miR-21 levels in the lung tissues of the COPD patients were significantly increased compared to the levels in the non-smokers by using RT-qPCR (Fig. 1A).The levels of Th17-related cytokines were quantified in the lung tissue homogenates of COPD patients and non-smokers using ELISA. IL-1β, IL-6, IL-17A, and RORγT protein expression, but IL-10 was significantly decreased and there were no changes in IL-21 and IL-22 between the COPD patients and non-smoking controls (4 out of 6 enrolled samples were randomly selected for western blot tests from control and COPD group respectively, Fig. 1B–G).

ROR-γT is Th17 related key transcription factor, Compared with non-smokers, the COPD patients had higher expression of ROR-γT in their lung tissues, which we confirmed using both RT-qPCR (Fig. 1H) and western blot analyses (Fig. 1I). These results suggest that there had high expression of both miR-21 and activation of Th17 cells in COPD patients.

### MiR-21 knockout alleviate COPD-related emphysema, lung functions and pulmonary inflammation changes

**Figure 2:**
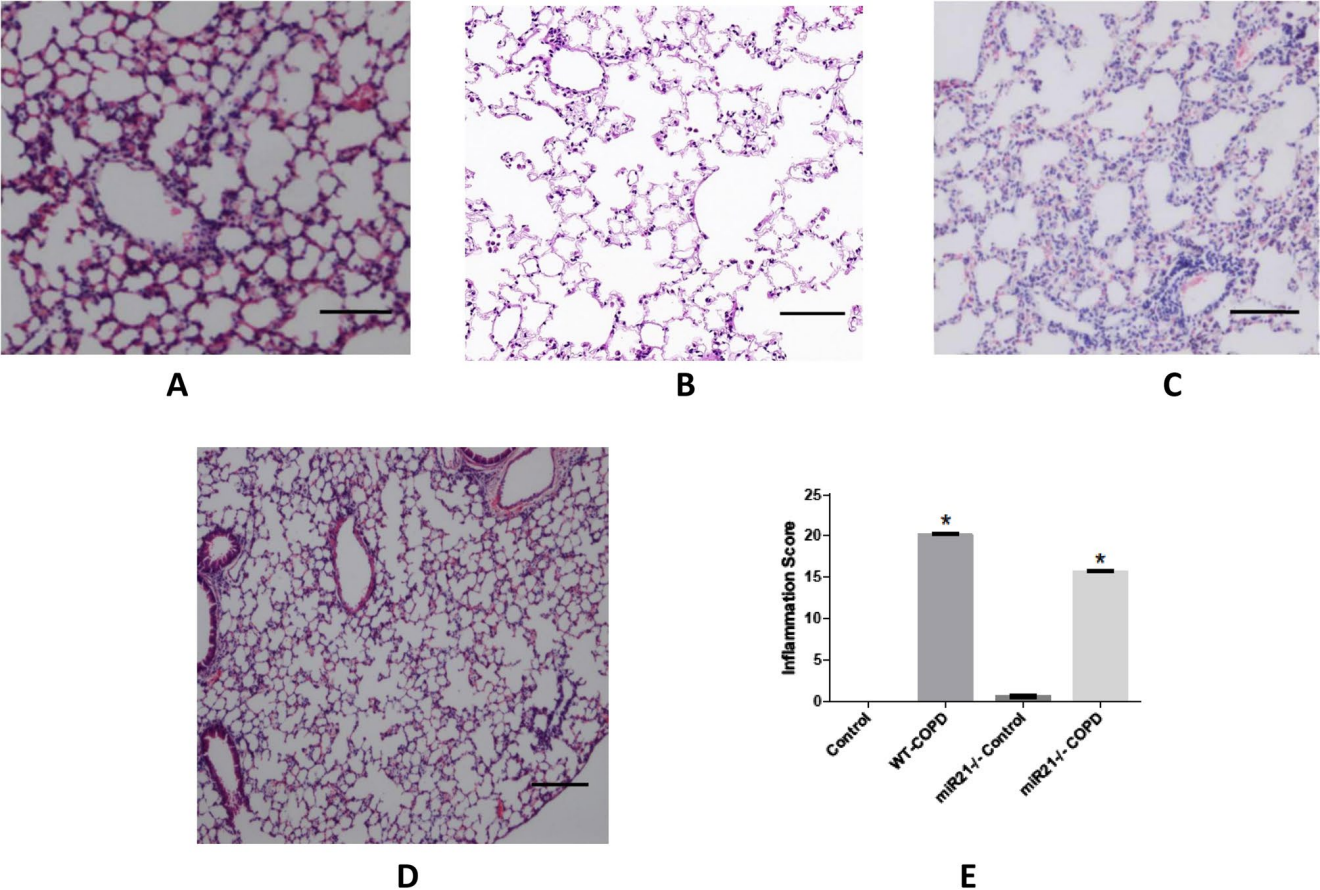
MiR-21 knockout alleviate COPD-related emphysema, lung functions and pulmonary inflammation changes. (**A**)–(**D**) H&E staining on paraffin-embedded mice lung tissue sections from control group, WT-COPD group, miR-21^−/−^ COPD group and miR-21^−/−^ control group respectively. The WT-COPD group exhibited enlarged alveolar space, thinner alveolar septum, and destroyed alveolar wall when compared with the control. Meanwhile, the miR-21^−/−^ COPD group had moderate pathological changes compared with the WT COPD group, but still worse than the control and the miR-21^−/−^ group have no notable changes when exposed to air. (**E**) The inflammation score of mice lung from different groups. The WT-COPD group had the highest inflammation score, followed by miR-21^−/−^ COPD group, and the miR-21^−/−^ control group was similar to WT control group. Magnification are all 400X, scale bar = 50 nm, The data are expressed as the mean ± SEM (n = 5 in each group). *: compared with the control group, P < 0.05; ^#^: compared with the WT COPD group, t-test and ANOVA test were applied in this part, P < 0.05.

We utilized H&E staining and IHC to evaluate the pathological changes of lung and the number of several inflammatory cells in lungs of mice from different groups. We observed that modeled-WT mice exhibited lowest MAST and highest MLI and DI% compared to the control group (Fig. 2A,B); and these changes in modeled miR-21^−/−^ mice were moderate, but still worse than control group (Fig. 2C). However, miR-21^−/−^ mice exposed to air showed no notable abnormal changes in lung tissue (Fig. 2D, Table 3).

**Table 3:** Morphological changes in the mouse lung following CS-exposure.

Groups	MAST (μm)	MLI (μm)	DI (%)
WT control	8.03 ± 0.18	28.44 ± 0.26	11.08 ± 0.46
WT CSE + CS	3.11 ± 0.48*	105.35 ± 0.82*	70.94 ± 3.66*
miR21^−/−^ control	8.23 ± 0.09	26.21 ± 0.16	12.23 ± 0.36
miR21^−/−^ CSE + CS	6.17 ± 0.64*^#^	48.79 ± 0.63*^#^	28.44 ± 0.74*^#^

Morphometric measurements of MAST (μm), MLI (μm) and DI (%) were performed in each group.

*P < 0.05 versus WT control group; ^#^P < 0.05 versusWT CSE + CS group (n = 5).

Moreover, modeled-WT mice showed the severest pathological changes as their RAW increased; Cdyn, PEF, and Ti/Te all decreased compared with the control. Modeled miR-21^−/−^ mice were also moderate, but still worse than the control, and meanwhile miR-21^−/−^ mice exposed to air showed no notable abnormal changes in lung function tests (Table 4).

**Table 4:** Comparison of lung function in different groups of mice.

Groups	Raw (cmH_2_O/ml/min)	Cydn (ml/cmH_2_O)	PEF (ml/s)	Ti/Te
WT control	0.54 ± 0.03	1.17 ± 0.01	2.01 ± 0.17	0.92 ± 0.05
WT CSE + CS	3.66 ± 0.05*	0.49 ± 0.01*	1.58 ± 0.06*	0.71 ± 0.05*
miR21^−/−^ control	0.62 ± 0.03	1.28 ± 0.02	1.95 ± 0.27	0.94 ± 0.02
miR-21^−/−^ CSE + CS	1.13 ± 0.001*^#^	0.82 ± 0.09*^#^	2.00 ± 0.29^#^	0.79 ± 0.03*^#^

Data are expressed as median and range.

*P < 0.05 versus control group, ^#^P < 0.05 versus WT CSE + CS group (n = 5).

Additionally, we analyzed the degree of inflammation in each group by using a histopathologic inflammatory scoring system as previously described^15^. As expected, the control mice scored the lowest, the modeled-WT mice scored the highest, and the modeled miR-21^−/−^ mice in the middle (Fig. 2E).

### MiR-21 activate the differentiation of Th17 cells

**Figure 3:**
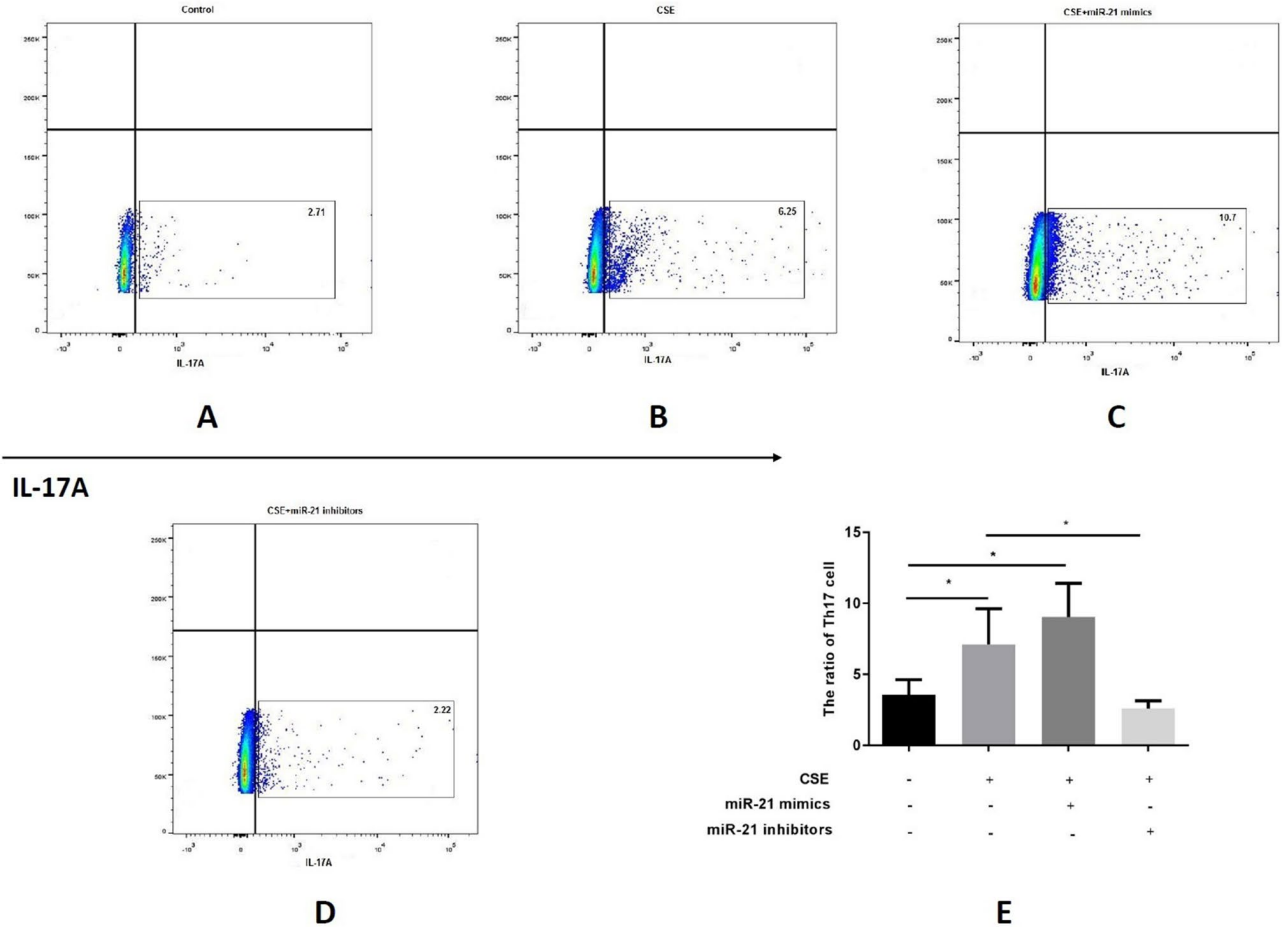
Th17 cell increased in COPD mice model and could be promoted by either CSE or miR-21. (**A**–**D**) Flow cytometry of IL-17A + cells in MACS-sorted CD4^+^  cells after different treatment (control, CSE, miR-21 mimics or miR-21 inhibitors respectively). (**E**) The quantification of IL-17A+ cells in CD4^+^ cells under different treatments. The CSE and miR-21 could both give rise the proportion of IL-17A + in CD4^+^  cells, meanwhile the miR-21 inhibitors work reversely. The data are expressed as the mean ± SEM (n = 5 in each group), t-test was applied in this part, *P < 0.05.

**Figure 4:**
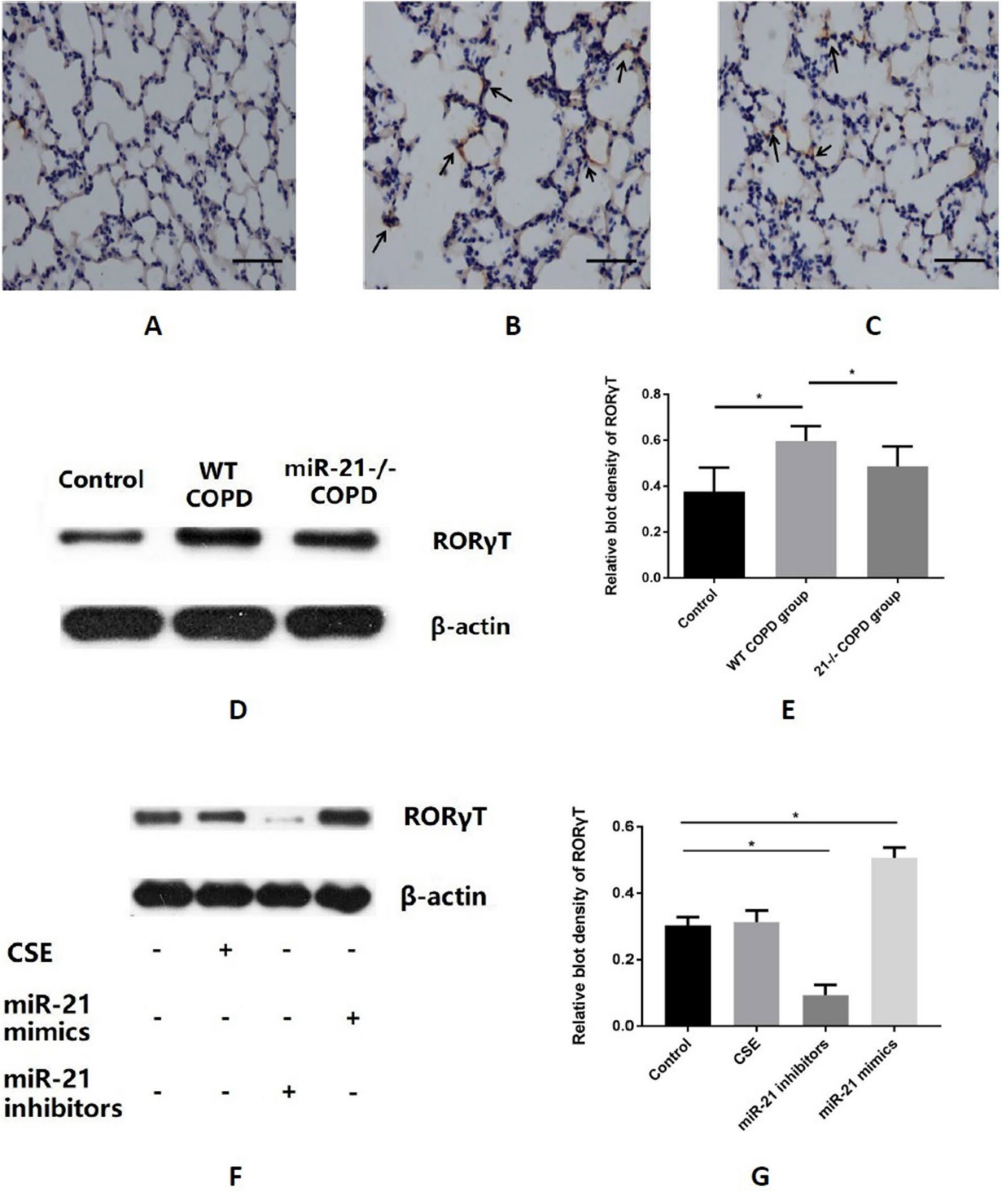
RORγT increased in COPD mice model and could be promoted by either CSE or miR-21. (**A**–**C**) IHC staining of RORγT in control, modeled-WT or miR-21^−/−^ mice lungs respectively. Modeled-WT mice have the highest RORγT in lungs while the miR-21^−/−^ mice are moderate but more than the control. (**D**) The western blot of RORγT in control, modeled-WT or miR-21^−/−^ mice lungs respectively. (**E**) The quantification of RORγT western blot density in different group. The, modeled-WT mice have the highest RORγT in lungs while the miR-21^−/−^ mice are moderate but more than the control. (**F**) The western blot of RORγT in control, CSE, miR-21 mimics or miR-21 inhibitors treated MACS-sorted CD4 + cells. (**G**) The quantification of RORγT western blot density in MACS-sorted CD4 + cells under different treatments. The CSE and miR-21 could both give rise the level of RORγT, meanwhile the miR-21 inhibitors work reversely. In western blot, different samples of the same marker are from the same blot. The data are expressed as the mean ± SEM (n = 5 in each group), t-test and ANOVA test were applied in this part, *P < 0.05.

We collected MACS-sorted CD4^+^ lymphocytes cells from the spleens of mice. Moreover, CSE, miR-21 mimics and inhibitors were added to different groups respectively. IL-17A was detected using flow cytometry to confirm the proportion of Th17 cells. Compared with the control group, CSE and miR-21 mimics further increased the proportion of Th17 cells while the miR-21 inhibitors worked reversely (Fig. 3A–E). RORγT is a crucial transcription factor of Th17 cells. We further measured the effects of miR-21 on RORγT by IHC in all mice from different groups. The highest level of RORγT was observed in the lung tissues of modeled-WT mice (Fig. 4B), followed by modeled miR-21^−/−^ mice (Fig. 4C), and the control the last (Fig. 4A). The western blot results (Fig. 4D,E) of RORγT level was consistent with the IHC. Additionally, RORγT expression in MACS-sorted splenic CD4^+^ cells from WT mice could also be increased by either CSE or miR-21 mimics, while the miR-21 inhibitors reduced it (Fig. 4F,G).

### MiR-21 partly activate Th17 cell differentiation partly through TGF-beta/Smad signaling pathway

**Figure 5:**
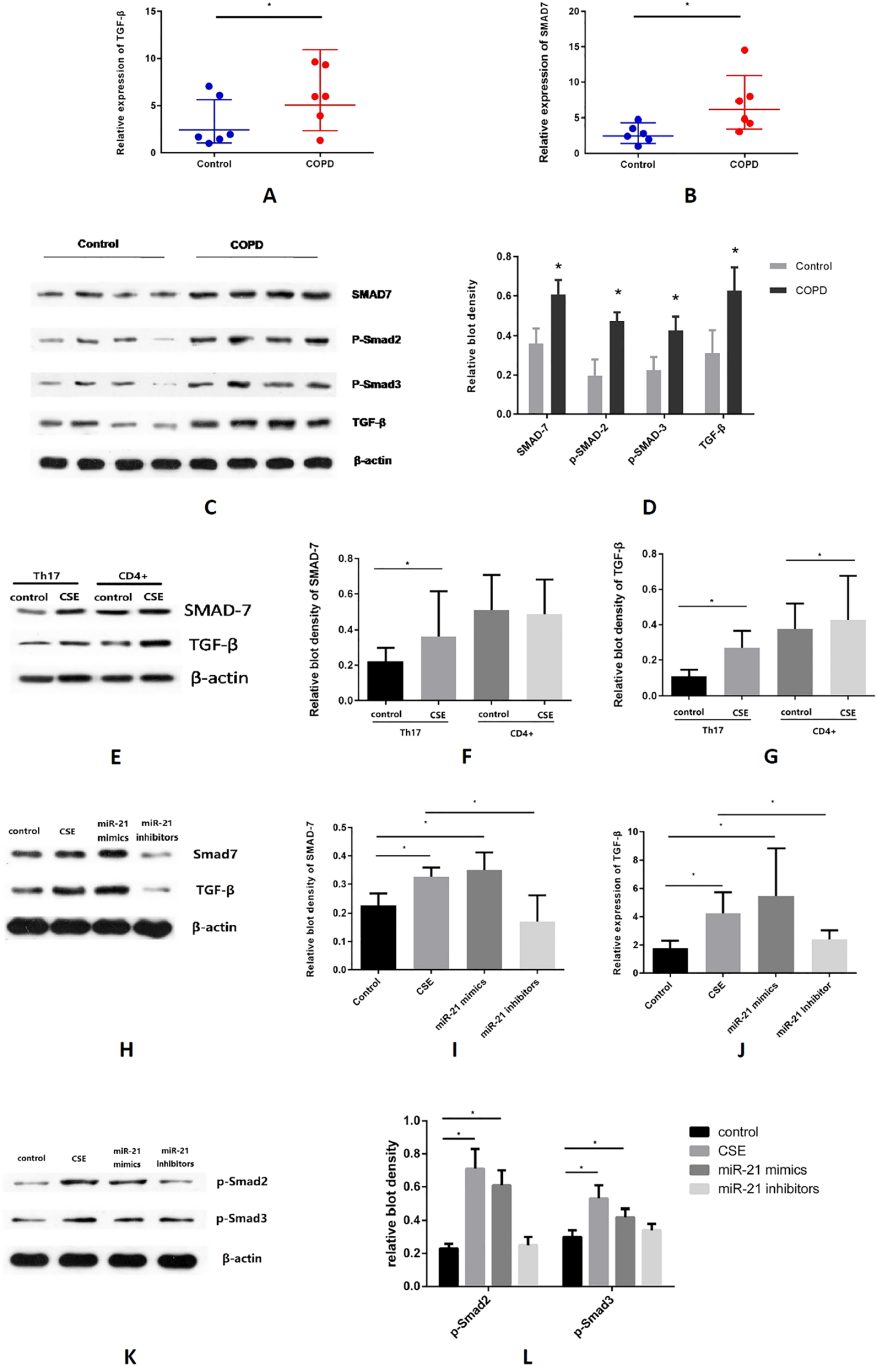
MiR-21 activate Th17 cell differentiation partly through TGF-beta/Smad signaling pathway. (**A**) TGF-β increased in lungs of COPD patients. (**B**) Smad7 increased in lungs of COPD patients. (**C**) The western blot of TGF-β/Smad pathway key factors in lungs of COPD patients. (**D**) COPD patients have higher level of all the TGF-β/Smad pathway key factors in lung. (**E**) The western blot of TGF-β and Smad7 in either Th17 cells or MACS-sorted CD4^+^ cells with the treatment of CSE. (**F**) SMAD7 increased significantly in Th17 cells but not in MACS-sorted CD4^+^ lymphocytes, after the treatment of CSE. (**G**) TGF-β increased significantly in both Th17 cells and MACS-sorted CD4^+^ cells, after the treatment of CSE. (**H**) The western blot of TGF-β and Smad7 in Th17 cells after the treatment of CSE, miR-21 mimics or miR-21 inhibitors. (**I**) Smad7 increased in Th17 cells after the treatment of both CSE and miR-21 mimics but decreased by miR-21 inhibitors. (**J**) TGF-β increased in Th17 cells after the treatment of either CSE or miR-21 mimics but decreased by miR-21 inhibitors. (**K**) The western blot of p-Smad2 and p-Smad3 in Th17 cells after the treatment of CSE, miR-21 mimics or miR-21 inhibitors. (**L**) p-Smad2 and p-Smad3 are both increased after the treatment of either CSE or miR-21 mimics but decreased by miR-21 inhibitors. In western blot, different samples of the same marker are from the same blot. The data are expressed as the mean ± SEM (n = 6 in each human lung sample group and n = 5 in each mice sample), t-test was applied in this part, *P < 0.05.

To investigate the potential mechanism of how miR-21 affect the functions of Th17 cell, we observed a significant increase of Smad7 and TGF-β mRNA levels in the lung tissues of the COPD patients (Fig. 5A,B). Western blot analysis showed that protein expression of Smad7, p-Smad2, p-Smad3, and TGF-β were increased in lung tissues of COPD patients compared to non-smokers (Fig. 5C,D). Furthermore, in Vitro, we found that the protein level of TGF-β, but not SMAD7, increased significantly in MACS-sorted CD4^+^ lymphocytes after the treatment of CSE, and moreover, TGF-β and Smad7 protein expressions were both significantly increased in Th17 cells following the treatment of CSE (Fig. 5E–G). Finally, we evaluated the roles of CSE and miR-21 on TGF-β/Smad signaling pathway by CSE, miR-21 mimics, or miR-21 inhibitors. CSE and miR-21 mimics increased the protein expression of p-Smad2, p-Smad3, Smad7, and TGF-β in Th17 cells, while the miR-21 inhibitors worked the other way around (Fig. 5H–L).

## Discussion

In the present study, we demonstrated that levels of Th17-related cytokines (IL-1β, IL-6, IL-17A, and TGF-α) and RORγT were increased in the lung tissues of COPD patients. We further found an increased Th17 cells proportion in the COPD mouse model. These results support previous findings from Wang and colleagues who demonstrated that the number of Th17 cells was increased in the peripheral blood of moderate-to-severe COPD patients, along with the levels of ROR-γT (an important transcription factor of Th17 cells), and many other inflammatory mediators, including IL-17A, IL-6, IL-21, IL-22, and IL-23. The levels of these inflammatory mediators have been shown to be negatively correlated with lung function^16^. Additionally, Imani and colleagues found that the levels of ROR-γT, IL-17A, and TGF-β1 were all increased in lung biopsies of COPD patients^17^. These studies were consistent with our present results, demonstrating a crucial role of Th17 cells and Th17-related cytokines in COPD pathogenesis.

Some studies suggested that miRNAs also play indispensable roles in the occurrences of many pulmonary diseases, such as lung cancer, COPD, asthma, and IPF^18^. In addition, Smigielska-Czepiel and colleagues found that miR-21 could interfere with the growth of activated memory T lymphocytes leading to apoptosis, indicating that miR-21 affects the activity of memory T lymphocytes^19^. Ando and colleagues confirmed that transfection of miR-21 into T lymphocytes could increase expressions of both TNF-α and IFN-γ^20^. By knocking down miR-21 in murine COPD models, we found that the levels of COPD-related lung pathological changes ameliorated, and this knockdown process would not affect the lung tissue structure when exposed to air. Further, we confirmed, using miR-21 mimics and inhibitors, that miR-21 could regulate Th17 cell differentiation and expression of the Th17 related transcription factor, ROR-γT. These results suggest that miR-21 plays an important role in COPD pathogenesis by regulating Th17 cell differentiation and ROR-γT expression.

TGF-β plays a crucial role in Th17 cell differentiation^21^. Specifically, overexpression of TGF-β1 has been shown to promote Th17 cell differentiation^13^. It has been hypothesized that miRNAs are co-regulators of this differentiation process. A previous study using a luciferase assay showed that miR-21 regulates the Smad/TGF-β pathway by suppressing Smad7^22^, and this discrepancy might be resulted from some other co-regulatory mechanisms by other pathways. Our results are similar to those in a study of Syed and colleagues, which showed a correlation between increases in the levels of Smad proteins and increased miR-21 expression in children with inflammatory bowel diseases^23^. Understanding exactly how different miRNAs co-regulate the Smad/TGF-β pathway, affecting Th17 cell differentiation and function, requires further investigations.

Smad proteins are important signal transduction molecules in the TGF-β superfamily^24^. Abnormal expression of molecules in the TGF-β/SMAD signaling pathway has been previously demonstrated in lung tissues from COPD patients^25^, and the changes in TGF-β precedes visible changes in lung pathology and function. The present study confirmed the abnormal expression of several key molecules in this pathway, under COPD pathogenesis. Moreover, we found the TGF-β/Smad pathway and the level of Th17 cells were both elevated under the stimulation of CSE, and these suggested that TGF-β/Smad pathway could at least contribute to the level of Th17 cells to some extents. And miR-21, which activates TGF-β/Smad pathway, could indirectly contribute to the Th17 cells differentiation. A further study of how miR-21 directly promote Th17 cells differentiation is necessary.

With the present study, there are some limitations merit consideration. Initially, we did not collect lung tissue from heavy smokers without COPD, for the reason that we just planned to make comparison between health people between confirmed COPD patient. However, with the data of heavy smokers could make this study more convincing as smoking is the leading cause of COPD. Further, we collected 6 human samples in each group but only showed 4 of them as representatives western blot results. Moreover, a further TGF-β/Smad pathway inhibitor experiment, comparing with the effects of miR-21 mimics/inhibitors, could contribute to the persuasion of the whole study.

To summarize, the present study, for the first time, demonstrated that CSE could give rise to miR-21 expression and promote Th17 cell differentiation, partly through the Smad/TGF-β pathway. Furthermore, an increase in Th17 cells could release numerous inflammatory cytokines which could further contribute to the developments of COPD. Additionally, inhibition of miR-21, either by genetic knockdown or miR-21 inhibitors, could be of great therapeutic value to COPD (Supplementary information).

## Supplementary Information

Supplementary Information.
