# Supplementary material for: The effects of the miR-21/SMAD7/TGF-β pathway on Th17 cell differentiation in COPD
Source: Sci Rep. 2021 Mar 18;11:6338. doi: 10.1038/s41598-021-85637-0 (PMC7973755; doi:10.1038/s41598-021-85637-0)
Supplement: Supplementary file 1 — Supplementary Information. [file 41598_2021_85637_MOESM1_ESM.docx]

**The effects of the miR-21/SMAD7/TGF-β pathway on Th17 cell differentiation in COPD**

Shengyang He^1^ MD, PhD, Shenghua Sun^1^ MD, PhD, Junjuan Lu^1^ MD, PhD, Lili Chen^1^ MD, Xiang Mei^1^ MD, Liqiu Li^1^ MD, Zhengpeng Zeng^1^ MD, Mubin Zhong^1^ MD, and Lihua Xie^1^ MD, PhD *

^1.^ Department of Pulmonary And Critical Care Medicine, The Third Xiangya Hospital Of Central South University, Changsha, China.

*Correspondence: Lihua Xie, [xyelyhua@163.com](mailto:xyelyhua@163.com)

FIGURE 1I
**
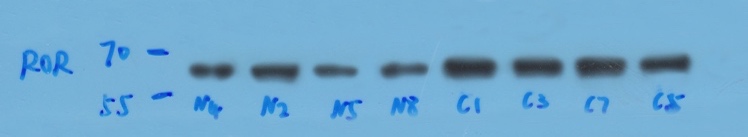

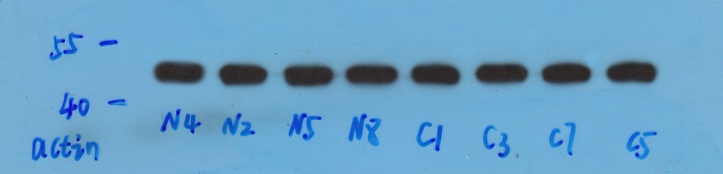
**

FIGURE 4D

**
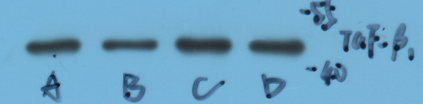
**

**
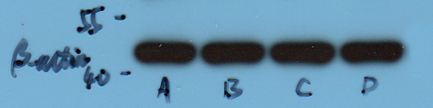
**

FIGURE 4F

**
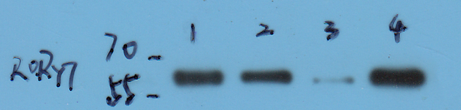
**

**
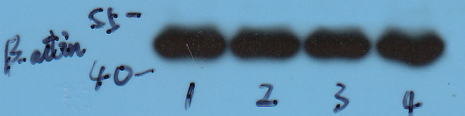
**

FIGURE 5C

**
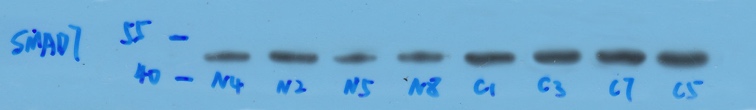
**

**
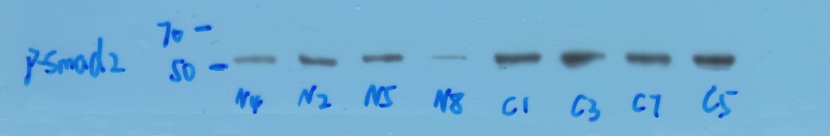
**

**
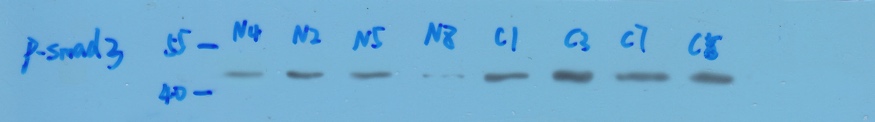
**

**
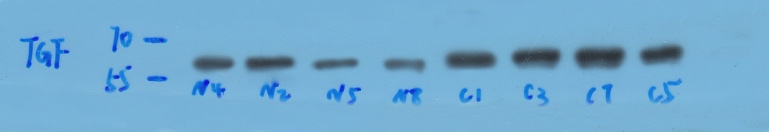
**

**
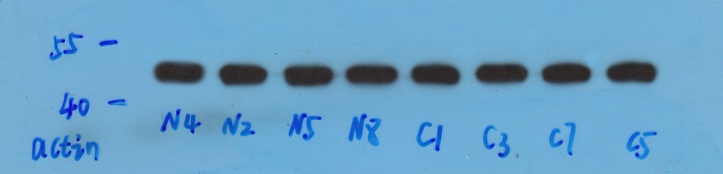
**

FIGURE 5E

SMAD-7
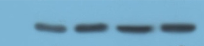


TGF-β
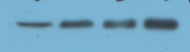


β-actin
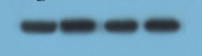


FIGURE 5H

**
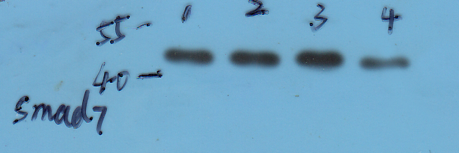
**

**
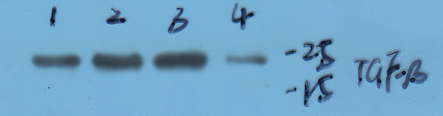
**

**
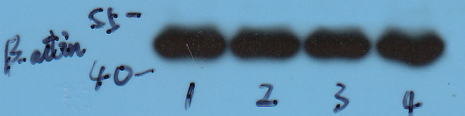
**

FIGURE 5K

**
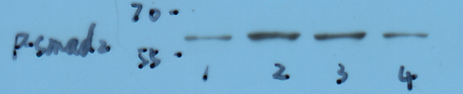
**

**
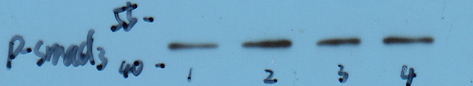
**

**
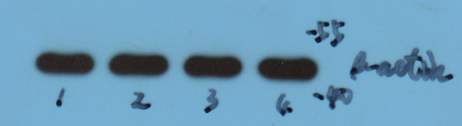
**
